# TLR4-Mediated Expression of Mac-1 in Monocytes Plays a Pivotal Role in Monocyte Adhesion to Vascular Endothelium

**DOI:** 10.1371/journal.pone.0104588

**Published:** 2014-08-12

**Authors:** Seung Jin Lee, Eun Kyoung Choi, Kyo Won Seo, Jin Ung Bae, So Youn Park, Chi Dae Kim

**Affiliations:** Department of Pharmacology, School of Medicine, and MRC for Ischemic Tissue Regeneration, Pusan National University, Yangsan, Gyeongnam, Republic of Korea; University of Nebraska Medical Center, United States of America

## Abstract

Toll-like receptor 4 (TLR4) is known to mediate monocyte adhesion to endothelial cells, however, its role on the expression of monocyte adhesion molecules is unclear. In the present study, we investigated the role of TLR4 on the expression of monocyte adhesion molecules, and determined the functional role of TLR4-induced adhesion molecules on monocyte adhesion to endothelial cells. When THP-1 monocytes were stimulated with Kdo2-Lipid A (KLA), a specific TLR4 agonist, Mac-1 expression was markedly increased in association with an increased adhesion of monocytes to endothelial cells. These were attenuated by anti-Mac-1 antibody, suggesting a functional role of TLR4-induced Mac-1 on monocyte adhesion to endothelial cells. In monocytes treated with MK886, a 5-lipoxygenase (LO) inhibitor, both Mac-1 expression and monocyte adhesion to endothelial cells induced by KLA were markedly attenuated. Moreover, KLA increased the expression of mRNA and protein of 5-LO, suggesting a pivotal role of 5-LO on these processes. In *in vivo* studies, KLA increased monocyte adhesion to aortic endothelium of wild-type (WT) mice, which was attenuated in WT mice treated with anti-Mac-1 antibody as well as in TLR4-deficient mice. Taken together, TLR4-mediated expression of Mac-1 in monocytes plays a pivotal role on monocyte adhesion to vascular endothelium, leading to increased foam cell formation in the development of atherosclerosis.

## Introduction

Monocyte adhesion to the endothelium in blood vessels is a key early event in the development and progression of atherosclerosis [1], [2], [3]. Monocytes, an important class of white blood cells, are known to contribute significantly to the development of atherosclerosis [4], [5]. They are actively recruited to atherosclerotic lesions, and promote plaque development by sustaining a chronic inflammatory reaction [6]. Recent evidence clearly demonstrates the role of toll-like receptors (TLRs) are known to mediate monocyte adhesion to EC via an increased expression of adhesion molecules on endothelial cells [7], [8], [9], however, its role on the expression of monocyte adhesion molecules is remain unknown.

TLR4 is expressed in monocytes [10], and TLR4 signaling appears to be a critical for the activation of inflammatory reaction in the monocytes [11], [12]. Recent studies have identified specific adhesion molecules in monocytes, such as, members of the β-2 integrin family, LFA-1 (CD11a/CD18), Mac-1 (CD11b/CD18), CD11c/CD18, and β-1 integrin, VLA-4 (CD49d/29), that interact with endothelial counter-ligands, such as, ICAM-1 or VCAM-1 [13], [14], [15], [16]. Furthermore, monocytes adhesion molecules, which are required for adhesion to endothelium, play important roles in the pathogenesis and progression of atherosclerosis [17], [18].

In our previous studies, we found that 5-LO is implicated in the development and progression of atherosclerosis [19], [20], [21]. KLA, a glycolipid component of the gram-negative bacterial cell wall, binds to the TLR4 on the surface of a variety of cells, including monocytes [22], [23], stimulates monocytes, and affects the productions of a number of inflammatory mediators, such as, 5-LO [24], [25]. Furthermore, the role played by TLR4 on the modulation of 5-LO suggests an important interaction between 5-LO-mediated inflammation and the development of atherosclerosis.

In this study, we determined functional role of TLR4 in endothelial adhesion of monocytes, and identified the involved mechanisms in *in vitro* studies. In *in vivo* studies, we investigated the potential role of TLR4 and Mac-1 on monocytes in endothelial adhesion of monocytes using wild-type control mice treated with an anti-Mac-1 antibody as well as TLR4-deficient mice. In addition, we also investigated the role of TLR4 signaling in 5-LO expression in monocytes.

## Methods

### Chemicals and antibodies

KLA (Kdo-Lipid A) was purchased from Adipogen (San Diego, CA, USA). Purified anti-human TLR4 antibody, anti-human Mac-1 antibody and anti-mouse IgG isotype control antibody were purchased from eBioscience (San Diego, CA). R-phycoerythrin (PE)-conjugated mouse anti-human Mac-1 (clone ICRF44; BD) antibody and PE-conjugated mouse IgG isotype control (clone MOPC-21) antibody were obtained from BD (San Diego, CA). Rabbit anti-mouse Mac-2 antibody was purchased from Abcam (Cambridge UK). MK-886 and various signal pathway inhibitors were purchased from EMD Serono, Inc. (Rockland, MA, USA) and Sigma-Aldrich Co. (Saint Louis, MO).

### Cell culture

THP-1 cells (a human monocytic leukemia cell line) were purchased from the ATCC (Manassas, VA, USA). Cells were grown in RPMI 1640 medium (Life Technologies) supplemented with 10% heat-inactivated fetal bovine serum (FBS), antibiotic-antimycotic, and L-glutamine (Life Technologies). Human Umbilical Vein Endothelial Cells (HUVEC) were obtained from Lonza Walkersville, Inc. (Walkersville, MD) and cultured in endothelial growth medium-2 (EGM-2 MV, Lonza). Cells were maintained at 37°C in a humidified 5% CO_2_/95% air atmosphere.

### Animals

TLR4 deficient mice (TLR4^(-/-)^) in the C57BL/6 background were kindly provided Dr. G. Y. Koh (KAIST, Korea) and wild-type (WT) control mice (C57BL/6) were purchased from Jackson Laboratories (Harlan Nossan, Italy). All the animals procedures were confirmed in accordance with the Guide for the Care and Use of Laboratory Animals published by the US National Institutes of Health (NIH Publication No. 85–23, revised 1996), and experimental protocols were approved by the Pusan National University Institutional Animal Care and Use Committee.

### Isolation of peripheral blood mononuclear cell (PBMC)

Peripheral blood from mice killed by cervical dislocation was collected by heart puncture into EDTA vacutainer tubes (Sarstedt, Newton, NC). PBMC were separated by density gradient centrifugation using Histopaque-1077 (Sigma) separating solution (Biochrom, Berlin, Germany). PBMC layers were collected and washed twice with Dulbecco's PBS and then resuspended at a concentration of 1×10^6^ cells/ml in RPMI 164 (GIBCO) containing 10% heat-inactivated FBS (Biochrom, Berlin, Germany) and 1% penicillin-streptomycin (Sigma-Aldrich Inc., St. Louis, MO).

### Flow cytometric analysis

To quantify Mac-1 expression, THP-1 cells were collected from cultures, washed with fluorescence-activated cell sorting (FACS) buffer (PBS containing 1% FCS and 0.05% NaN_3_), first incubated with FcR blocker (anti-human IgG; Sigma-Aldrich Co.) to block nonspecific antibody binding, and bound with PE-conjugated mouse anti-Human CD11b/Mac-1 (clone ICRF44; BD) with matched pairs of PE-conjugated mouse IgG, isotype antibody. The analysis was performed using FACSCalibur and CELLQUESTPRO software (BD).

### Adhesion assay

THP-1 cells were labeled with 0.2 mg/L calcein-AM for 30 min at 37°C, and seeded onto confluent HUVEC. After 2 h, co-cultured cells were washed with 1× PBS containing 1% bovine serum albumin (BSA), and images were obtained using an inverse optical microscope (Axiovert 25) and Axio Vision Release 4.7 software (Carl Zeiss MicroImaging GmbH, Oberkochen, Germany). Localization data was quantified using the Metamorph image analysis system (Molecular Devices, LLC, Downingtown, PA, USA).

### New enface method for optimal observation of endothelial surface (NEMOes)

Mice were sacrificed by cervical dislocation. Fixation and tissue preparation were performed by systemic perfusion at a pressure of 180 cm H_2_O via the left ventricle using 500 ml of normal saline followed by 300 ml of 10% buffered formalin. After fixation, aortas were dissected carefully from aortic arches to the lower thoracic region and immersed in 10% buffered formalin overnight at 4°C. Aorta was divided into 8–12 mm long segments, proximal ends of segments were marked, and segments were then placed in 0.05% hydrogen peroxide in methanol for 20 min at room temperature. After rinsing three times with PBS, segments were placed in boiling citrate buffer, and cooled gradually under running water. The segments were incubated with rabbit anti-mouse Mac-2 monoclonal antibody and then with biotinylated goat anti-Rabbit IgG. Finally, they were reacted with horseradish peroxide-conjugated streptavidin (Dako).

### RNA isolation and RT-PCR

Total RNA was isolated using TRIzol reagent (Life Technologies), and reverse transcribed with the Improm II reverse transcription system. The reverse transcribed cDNA was amplified by PCR using specific primers and conditions, as previously described [26]. PCR products were separated on 1.2% agarose gels and stained with EtBr solution.

### Western blot analysis

To determine the expression of 5-LO, THP-1 cells were washed with ice-cold PBS and lysed in lysis buffer. Cellular proteins were resolved by 10% sodium dodecyl sulfate-polyacrylamide gel electrophoresis and transferred to polyvinylidene difluoride membranes. After blocking membranes with 1× TBST containing 5% skim milk, they were probed with an anti-5-LO (1∶1,000) or anti-beta actin antibody (1∶10,000). After a second incubation with secondary antibodies, according to the manufacturer's instructions, immune complexes were then detected with ECL reagent.

### Statistical analysis

Results were expressed as means ± SEM. Significances were determined using the Student's t-test for unpaired observations between two groups or by ANOVA with Bonferroni's correction for more than two groups. Statistical significance was accepted for *p* values <0.05.

## Results

### Role of TLR4 on adhesion molecule expression in monocytes

**Figure 1:**
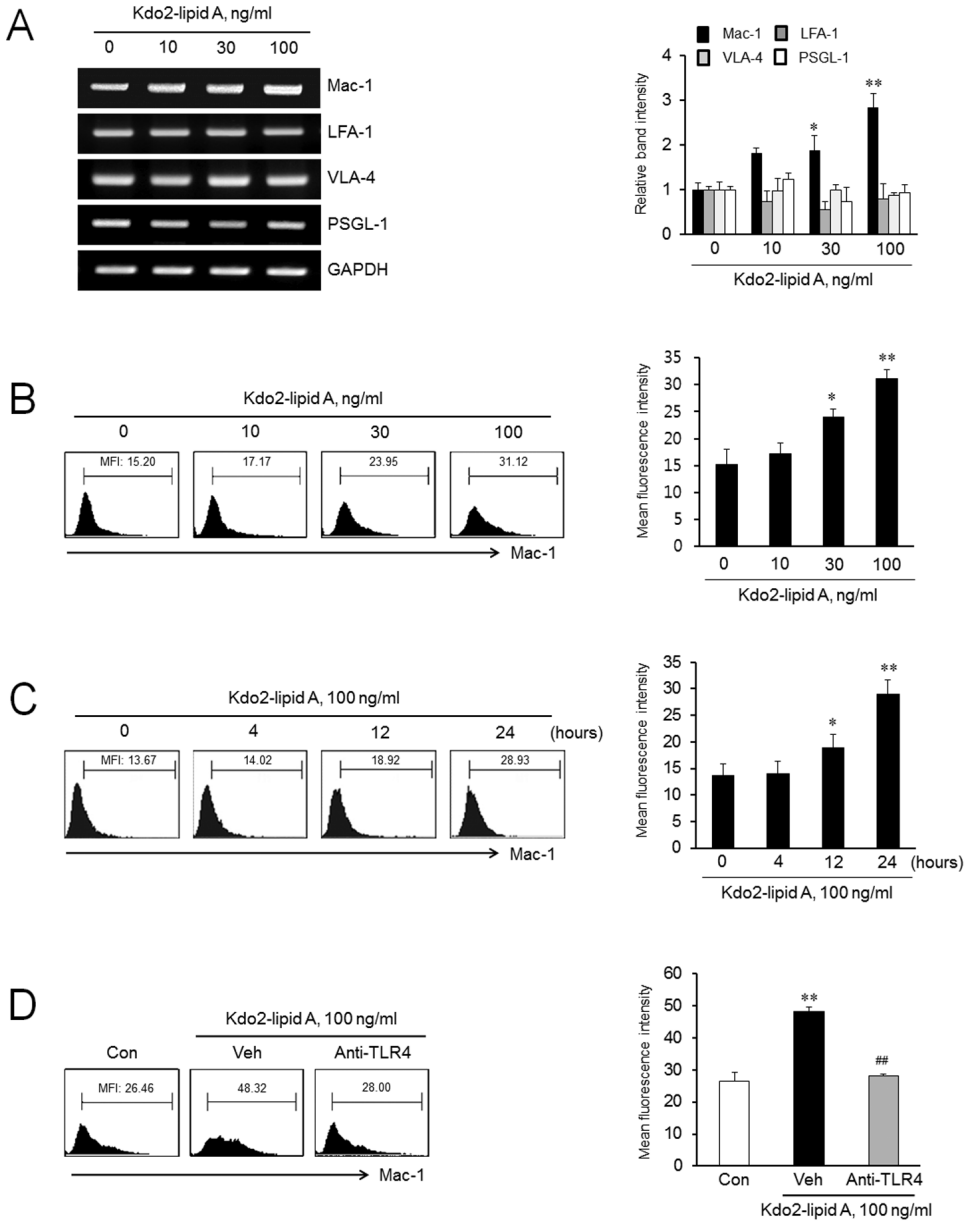
Role of TLR4 on adhesion molecule expression in monocytes. (A) THP-1 monocytes were treated with the indicated concentrations of Kdo2-lipid A (KLA) for 8 h, and total RNA isolated from cells was analyzed for mRNA expression of adhesion molecules, including Mac-1, LFA-1, VLA-4, and PSGL-1 by RT-PCR. (B) Cells were stimulated with KLA for 24 h, and then analyzed by flow cytometry for Mac-1 protein expression using R-phycoerythrin (PE)-conjugated anti-Mac-1 antibody. The surface expression of Mac-1 protein on monocytes was represented as mean fluorescent intensity (MFI). (C) Cells were stimulated with KLA for the indicated time, and then analyzed by flow cytometry for Mac-1 protein expression. (D) Monocytes were pretreated with an anti-TLR4 antibody (10 µg/ml) for 1 h, stimulated with KLA (100 ng/mL) for 24 h, and then Mac-1 protein expression was measured by flow cytometry. Right panels represent quantitative data for left panels, and represent means ± SEM (n = 4–7). *p<0.05, **p<0.01 vs. value at concentration 0 or time 0 or control (Con). *##p<*0.01 vs. vehicle (Veh).

To determine the effects of TLR4 on adhesion molecule expression in monocytes, we examined mRNA expression of various adhesion molecules in monocytes that were stimulated with KLA, a TLR4 agonist. As shown in Fig. 1A, KLA-stimulated monocytes greatly increased Mac-1 mRNA expression in a concentration-dependent manner, but not others including LFA-1, VLA-4, and PSGL-1. Consistent with the upregulation of Mac-1 mRNA expression, flow cytometric analysis also demonstrated concentration- and time-dependent increases in Mac-1 protein expression in KLA-treated cells (Fig. 1B and 1C). To further investigate whether the effects of KLA-induced Mac-1 expression was mediated via the TLR4, THP-1 monocytes were pretreated with a functional blocking antibody against TLR4, and then stimulated with KLA. As shown in Fig. 1D, the KLA-induced Mac-1 expression was significantly inhibited by pretreatment with a TLR4 antibody, demonstrating the involvement of TLR4 in KLA-induced Mac-1 expression in monocytes.

### Role of Mac-1 on TLR4-mediated monocyte adhesion to endothelial cells

**Figure 2:**
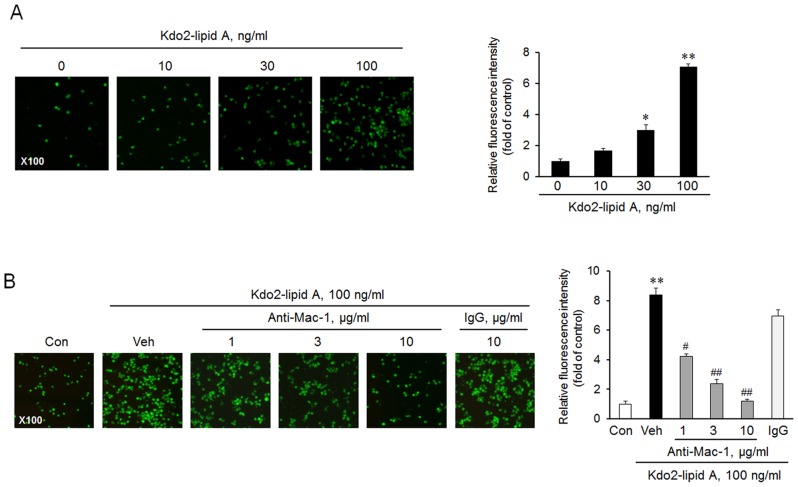
Functional role of Mac-1 on monocyte adhesion to endothelial cells. (A) THP-1 monocytes were stimulated with the indicated concentrations of KLA for 24 h, and labeled with calcein-AM for 30 min, and then co-cultured with HUVEC for 2 h. (B) Monocytes were pretreated with anti-Mac-1 antibody for 1 h, stimulated with 100 ng/mL KLA for 24 h, and then co-cultured with HUVEC for 2 h. Monocytes adhered to HUVEC were visualized by fluorescence microscopy at 100× magnification. Fluorescent intensity was analyzed using a MetaMorph microscope image analysis system, and represented as means ± SEM (n = 4–6). IgG was used for the negative control for anti-Mac-1 antibody. *p<0.05, **p<0.01 vs. value at concentration 0 or control (Con). #*p*<0.05, ##*p*<0.01 vs. vehicle (Veh).

To investigate the functional role of Mac-1 on monocytes in monocyte adhesion to vascular endothelium, we performed an adhesion assay using THP-1 monocytes and HUVEC. As shown in Fig. 2A, KLA increased monocyte adhesion to HUVEC in a concentration-dependent manner. The KLA-induced monocyte adhesion to HUVEC was significantly inhibited in a concentration-dependent manner by an anti-Mac-1 antibody (Fig. 2B), demonstrating the involvement of Mac-1 on monocytes in TLR4-mediated adhesion of monocytes to endothelial cells.

### Role of TLR4 in monocyte adhesion to vascular endothelium

**Figure 3:**
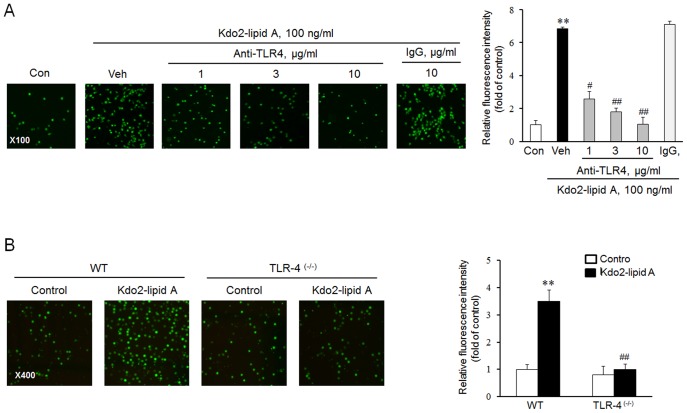
Role of TLR4 on monocyte adhesion to endothelial cells. (A) THP-1 monocytes were pretreated with various concentrations of anti-TLR4 antibody for 1 h, stimulated with KLA for 24 h, and then co-cultured with HUVEC for 2 h. Monocytes adhered to HUVEC were visualized by fluorescence microscopy, and quantified intensity was represented as means ± SEM (n = 3–5). IgG was used for the negative control for anti-TLR4 antibody. **p<0.01 vs. control (Con), #*p<*0.05, *##p<*0.01 vs. vehicle (Veh). (B) Primary monocytes isolated from peripheral blood (PBMC) of wild-type (WT) and TLR4^(-/-)^ mice were stimulated with KLA, and monocytes adhered to HUVEC were visualized by fluorescence microscopy. Right panels represent quantitative data for left panels, and represent means ± SEM (n = 3–5). **p<0.01 vs. control, ^##^ *p*<0.01 vs. corresponding value in WT.

To investigate the functional role of TLR4 in monocyte adhesion to endothelial cells, we performed an adhesion assay using THP-1 cells and primary monocytes isolated from peripheral blood of mice (PBMC). As shown in Fig. 3A, KLA-induced monocyte adhesion to HUVEC was significantly attenuated by pretreatment with anti-TLR4 antibody in a dose-dependent manner. In *ex vivo* studies, the endothelial adhesion of PBMC from KLA-treated WT mice was markedly increased, which was attenuated in PBMC isolated from TLR4^(-/-)^ mice (Fig. 3B). These results indicated that TLR4 plays a key role in monocyte adhesion to endothelium by KLA in *in vitro* and *ex vivo* studies.

### Role of 5-LO in TLR4-mediated adhesion of monocytes to endothelial cells

**Figure 4:**
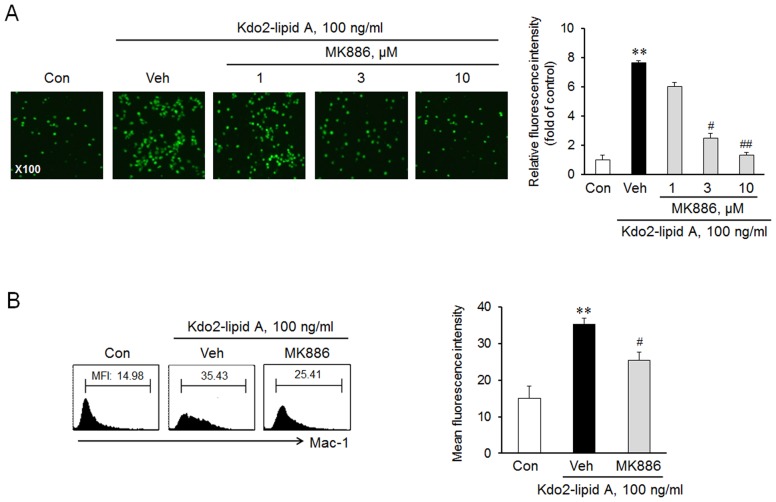
Involvement of 5-LO in TLR4-mediated monocyte adhesion to endothelial cells. (A) Monocytes were pretreated with the indicated concentrations of MK886, a 5-LO inhibitor, for 1 h, and then stimulated with 100 ng/mL KLA for 24 h. Calcein AM-labeled monocytes were co-cultured with HUVEC for 2 h, and cells adhered to HUVEC were visualized by fluorescence microscopy at 100× magnification. (B) The surface expression of Mac-1 protein on KLA-stimulated monocytes was analyzed by flow cytometry. Right panels represent quantitative data for left panels, and represent means ± SEM (n = 4–6). **p<0.01 vs. control (Con), #*p<*0.05, *##p<*0.01 vs. vehicle (Veh).

To investigate the link between TLR4 and 5-LO pathways, we determined the role of 5-LO in TLR4-mediated adhesion of monocytes to endothelial cells. In this study, THP-1 monocytes were pretreated with MK886, a 5-LO inhibitor, and then stimulated with KLA for 24 h. As shown in Fig. 4A, the KLA-induced monocyte adhesion to HUVEC was markedly decreased by pretreatment with MK886 in a concentration-dependent manner. Futhermore, KLA-induced expression of Mac-1 protein was also inhibited by pretreatment with MK886 (Fig. 4B), suggesting that 5-LO plays a key role in the TLR4-mediated monocyte adhesion to endothelial cells via an increased expression of Mac-1 on monocytes.

### Role of TLR4 in 5-LO expression in monocytes

**Figure 5:**
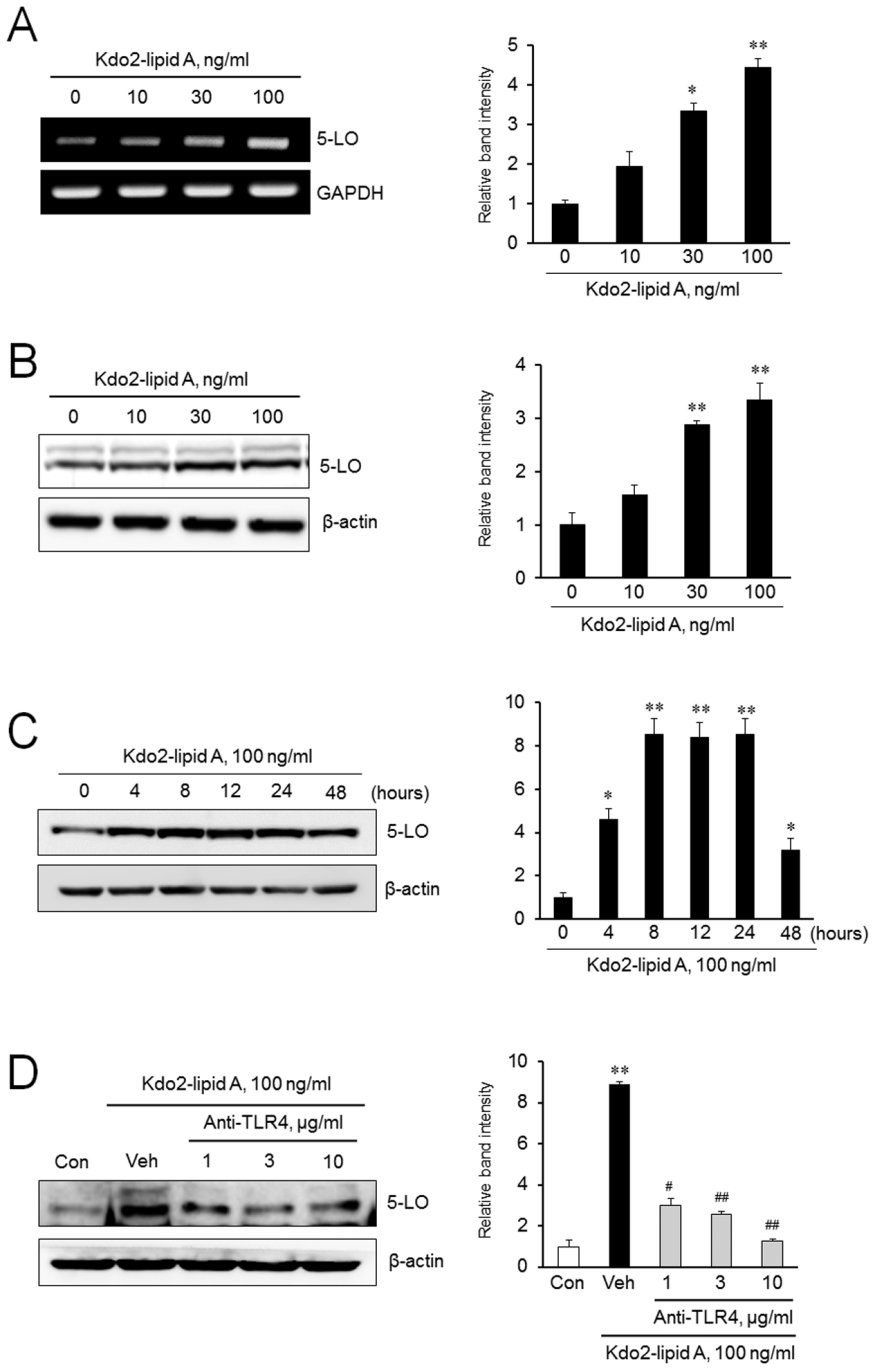
Role of TLR4 on 5-LO expression in monocytes. THP-1 monocytes were treated with indicated concentrations of KLA, and the expression of 5-LO mRNA (A) and protein (B) was analyzed by RT-PCR and immunoblotting, respectively. (C) Cells were stimulated with KLA (100 ng/ml) for the indicated time, and then 5-LO protein expression was analyzed by immunoblotting. (D) Cells were pretreated with various concentrations of anti-TLR4 antibody for 1 h, stimulated with KLA for 24 h, and then 5-LO protein expression was analyzed by immunoblotting. Right panels represent quantitative data for left panels, and represent means ± SEM (n = 4–6). *p<0.05, **p<0.01 vs. value at concentration 0 or time 0 or control (Con). #p<0.05; ##*p*<0.01 vs. vehicle (Veh).

To determine the role of TLR4 in 5-LO expression, THP-1 monocytes were stimulated with various concentrations of KLA. As shown in Fig. 5A and 5B, 5-LO mRNA and protein expression were increased significantly by KLA in a concentration-dependent manner. In the time-course study, 5-LO expression in monocytes started to increase at 4 h after KLA treatment, and then an increased expression of 5-LO was maintained up to 24 h (Fig. 5C). When monocytes were pretreated with anti-TLR4 antibody, the KLA-induced 5-LO expression was significantly attenuated in a concentration dependent manner (Fig. 5D), suggesting a pivotal role of TLR4 on KLA-induced 5-LO expression in monocytes.

### Role of Mac-1 and TLR4 in monocyte adhesion to aortic endothelium in *in vivo* study

**Figure 6:**
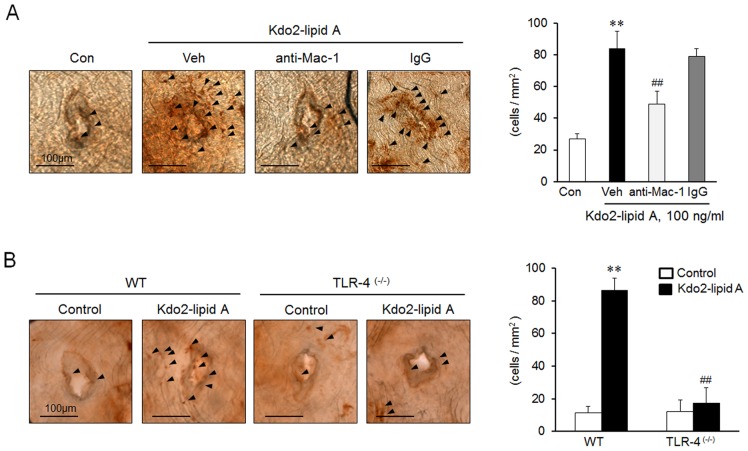
Role of Mac-1 on TLR4-mediated monocyte adhesion to vascular endothelium. (A) Wild-type (WT) mice were pretreated with anti-Mac-1 or control antibody (IgG) for 4 h, and then treated with KLA (3 µg/mouse) for 24 h. (B) WT and TLR4 ^(-/-)^ mice were treated with KLA (3 µg/mouse) for 24 h. Endothelial layer of isolated aorta was immunostained with anti-Mac-2 antibody for visualization of monocytes adhered to the aortic endothelium. Arrowheads denote Mac-2-positive cells. Scale bar  = 100 µm. Right panels represent quantitative data for left panels, and represent means ± SEM (n = 4–6). **p<0.01 vs. control (Con), ^##^ *p*<0.01 vs. vehivle (Veh) or corresponding value in WT.

In *in vivo* system, we stained endothelial layer with anti-Mac-2 antibody as a marker for the presence of monocytes and determined monocytes adhered to endothelial surface surrounding the orifice of aorta. The number of monocytes adhering to the aortic endothelium was significantly increased in KLA-treated mice relative to vehicle-treated mice, which was significantly inhibited by pretreatment with anti-Mac-1 antibody (Fig. 6A). We also determined the functional role of TLR4 in monocyte adhesion to aortic endothelium using TLR4^(-/-)^ mice. As shown in Fig. 6B, KLA increased monocyte adhesion to aortic endothelium of wild-type (WT) mice, which was markedly attenuated in TLR4-deficient mice. These results indicate that Mac-1 plays a key role in TLR4-mediated monocyte adhesion to aortic endothelium in *in vivo* system.

## Discussion

This study investigated the role of TLR4 in monocytes in Mac-1-mediated endothelial adhesion of monocytes. The results of our *in vitro* study showed that KLA, a specific TLR4 agonist, enhanced monocyte adhesion to HUVEC, which was mediated by an increased expression of Mac-1 on monocytes. In addition, the increased expression of Mac-1 by KLA has been demonstrated to be regulated by an increased expression of 5-lipoxygenase (LO) in monocytes. These results were also confirmed in *in vivo* studies, in which monocytes adhering to aortic endothelium was significantly increased in KLA-treated mice than in controls, which was significantly inhibited by pretreatment with an anti-Mac-1 antibody as well as in TLR4^(-/-)^ mice. These results support the hypothesis that TLR4 in monocytes contributes Mac-1-mediated monocyte adhesion to vascular endothelium, which is mediated by an increased expression of 5-LO in monocytes.

TLR4 has been shown to be an important factor responsible for triggering atherosclerosis and the associated cardiovascular diseases [27], [28]. In particular, activation of TLR4 by specified ligands can provoke vascular inflammation linked to atherosclerosis [11], [12]. The modulation of monocyte adhesion to vascular endothelium, which is one of the earliest detectable cellular responses and processes leading to vascular inflammation, could be an important target for preventing the development and progression of atherosclerosis [3], [6]. Recent evidence clearly demonstrates the role of TLR4 in monocyte adhesion to vascular endothelium via an increased expression of adhesion molecules on endothelium [8], [9], however, its precise role on the expression of monocyte adhesion molecules remains unknown. Thus, this study investigated the role of TLR4 in monocytes on the adhesion molecules expression, and determined the functional role of TLR4-induced adhesion molecules on monocyte adhesion to vascular endothelium.

The adhesion of circulating monocytes occurs through a tightly regulated multistep process that is mediated by a combination of cell surface adhesion molecules [29], [30]. Our results support the importance of monocytes in endothelial adhesion with regard to the utilization of adhesion molecules on monocytes for an increased adhesion of monocytes to vascular endothelium. In the present study, KLA, a TLR4 ligand, enhanced the expression of Mac-1 on monocytes in concentration- and time-dependent manners. Thus, it is considered that KLA-induced cell adhesion appears to be resulted from the upregulation of Mac-1 surface expression in monocyte cells, making a firm association with ICAM-1 on vascular endothelium [13], [14]. This hypothesis was confirmed by our *in vitro* studies, in which monocyte adhesion to HUVEC was inhibited by pretreatment with an anti-Mac-1 antibody. Moreover, KLA stimulated monocyte adhesion to HUVEC in a concentration-dependent manner, which was significantly inhibited by pretreatment with an anti-TLR4 antibody. Thus, it is suggested that TLR4 signaling in monocyte might be a major contributor in the initiation of monocyte recruitment. Considering the facts that endothelial cells in artery [31] *and* postcapillary venules [32] express high levels of ICAM-1 and VCAM-1 which bind Mac-1 on monocytes, changes in monocytes during TLR4 stimulation might also be important in mediating the toxic effects of bacteria in chronic infections as well as in the initiation of vascular inflammation.

On the basis of the reports that 5-LO signaling is associated with multiple inflammatory conditions including atherosclerosis [33], [34], [35], our results provide an important insight into the mechanism of TLR4 signaling in the modulation of 5-LO expression in monocytes. In the present study, KLA induced monocytes adhesion to HUVEC via an increased expression of Mac-1 on monocytes, which was attenuated by the inhibition of 5-LO pathways with MK886, a 5-LO inhibitor. These results suggest that 5-LO might be involved in monocyte adhesion to vascular endothelium through an increased expression of Mac-1. To evaluate the role of TLR4 on the regulation of 5-LO expression, we stimulated monocyts with KLA, and then 5-LO mRNA and protein expression were determined. As shown in Fig. 5, KLA enhanced expression of 5-LO mRNA and protein. The KLA-induced 5-LO expression was significantly attenuated by pretreatment an anti-TLR4 antibody, demonstrating the role of TLR4 signaling in the regulation of 5-LO expression in monocytes.

Finally, these *in vitro* results were confirmed in *in vivo* studies using control mice treated with an anti-Mac-1 antibody and TLR4-deficient mice. In the present study, we examined the effect of Mac-1 on the monocyte adhesion to the endothelial surface surrounding the orifice of aorta in mice. The number of monocytes adhered to the aortic endothelium was significantly increased in KLA-treated mice compared to that in vehicle-treated mice, which was significantly attenuated by pretreatment with the functional blocking antibody against Mac-1. Moreover, using TLR4-deficient mice, we demonstrated the pivotal role of TLR4 in KLA-induced monocyte adhesion to aortic endothelium. These results suggest a pivotal role of TLR4 in Mac-1 mediated adhesion of monocytes to vascular endothelium. Thus, it is suggested that TLR4 signaling in monocyte might be a major contributor in the initiation of atherosclerosis.

In summary, our study demonstrated that TLR4 mediated monocyte adhesion to vascular endothelium through an increased expression of Mac-1 on monocytes, and this was modulated by an increased expression of 5-LO. In addition, the importance of TLR4 and Mac-1 on monocytes in endothelial adhesion of monocytes was also demonstrated in *in vivo* studies, in which monocyte adhesion to endothelial surface of aorta was markedly attenuated in mice treated with an anti-Mac-1 antibody as well as in TLR4-deficient mice. Our results suggest that TLR4/5-LO/Mac-1 signaling in monocytes might be a major contributor in the initiation of vascular inflammation.
